# The Knowledge, Attitude, and Perception of Needlestick Injuries Among Dental Students in Riyadh, Kingdom of Saudi Arabia: A Cross-Sectional Survey

**DOI:** 10.7759/cureus.50939

**Published:** 2023-12-22

**Authors:** Abdul Salam Thekkiniyakath Ali, Nasser Alsour, Abdullah S Almansour, Ahmed Albahlal, Haitham Alahmari, Faisal Alrumi, Saleh Alhellal

**Affiliations:** 1 Preventive Dental Sciences Department, College of Dentistry, King Saud bin Abdulaziz University for Health Sciences, Riyadh, SAU

**Keywords:** hepatitis, attitude, knowledge, needle injury, needle stick injury

## Abstract

Introduction: Needlestick injuries (NSIs) represent a significant source of bloodborne viruses in the healthcare sector, particularly in dentistry. Developing effective infection control programs necessitates access to surveillance data. A comprehensive evaluation, encompassing human immunodeficiency virus (HIV), hepatitis B virus (HBV), hepatitis C virus (HCV), and serological testing, is crucial due to the potential lack of immediate symptoms in infected patients. It is essential to verify hepatitis B and tetanus immunization statuses and seek guidance from infectious disease experts for post-exposure prophylaxis.

Aim and objective: This study aims to assess the knowledge, attitudes, and perceptions of NSIs among dentistry students in Riyadh, Saudi Arabia.

Methods: Conducted during the 2023-2024 academic year in Riyadh, Saudi Arabia, this cross-sectional study evaluated the knowledge, attitudes, and perceptions of undergraduate dental medicine students regarding NSIs. Ethical approval was obtained, and informed consent was meticulously collected. Before participation, potential subjects were furnished with a comprehensive information sheet outlining the study's purpose, the survey's nature, expected duration, and potential risks or benefits. The document emphasized their unequivocal right to withdraw from the study at any point without any adverse consequences. Data collection relied on an online survey administered to third and final-year dental students selected from specific universities. This selection criterion ensured that participants were directly relevant to the dental education context under investigation. Careful exclusions were made to minimize potential bias, particularly by excluding students from academic years other than the third and final years, thus maintaining a specific focus on this subgroup. Data analysis primarily compared NSI occurrences and awareness based on dental college and academic year. Detailed findings are presented in the results section.

Results: The study unveiled high vaccination rates (95.23%) and significant levels of education regarding bloodborne infections (81.38%) among participants. However, it was noteworthy that 72.72% believed their vaccinations offered complete protection. Only 47.18% believed that wearing surgical gloves reduced the risk of NSIs, while a substantial majority (93.07%) opposed the practice of needle recapping. Moreover, 76.19% demonstrated knowledge of post-exposure prophylaxis, indicating room for improvement in healthcare safety practices. Statistical analysis identified significant associations between the Dental College attended and both NSI occurrence (χ²=12.164, p=0.058) and awareness (χ²=14.629, p=0.023). Conversely, the academic year exhibited no significant relationship with either NSI occurrence (χ²=1.2, p=0.55) or awareness (χ²=0.44, p=0.8). Additionally, the study revealed non-normal distributions for both NSI occurrence (p<0.001) and awareness (p<0.001) among participants.

Conclusion: In conclusion, this study underscores the pivotal role of awareness in mitigating NSI occurrences among dental students. Irrespective of their academic year, heightened awareness significantly correlated with reduced NSI incidence. These findings bear significant implications for dental education and practice, emphasizing the imperative need for comprehensive education and awareness initiatives to bolster healthcare safety among dental professionals.

## Introduction

A needlestick injury (NSI) refers to an inadvertent puncture wound brought about by a hollow-bore needle (or similar sharp item) containing another person's blood or body fluid. A sharps injury (SI) refers to a puncture wound that occurs when the skin is penetrated by sharp instruments or as a result of mishaps that take place within a medical environment [1].

The oral cavity serves as a conducive habitat for the spread of numerous infectious organisms, such as dangerous bloodborne viruses, as well as for their inoculation as well as reproduction, including human immunodeficiency virus (HIV), hepatitis B virus (HBV), severe acute respiratory syndrome coronavirus 2 (SARS-CoV-2). The heightened susceptibility of dental practitioners to infectious diseases can be attributed to several factors, including restricted visibility and accessibility, frequent utilization of aerosol-generating devices, and close proximity between dentists and patients during treatment operations [2]. The prevalence of HBV infection among dentists was shown to be significantly greater compared to the general population on a global scale. Specifically, in the United States, the infection rate among dentists was six times higher, while in Germany it was four times higher, and in Japan, it was 2.5 times higher. Various methods for the transmission of infectious microorganisms have been identified in the context of dental care delivery. These routes include (1) contact directly with oral fluids, blood, or other materials from the patient; (2) indirect interaction with objects that have been contaminated; (3) contact of the oral mucosa with droplets, nasal, or conjunctival expelled by an infected individual through actions like sneezing, coughing, talking, or while using dental instruments; (4) inhaling the airborne microorganisms [3,4].

NSIs have been identified as a significant contributor to the spread of pathogenic viruses, primarily through either indirect or direct transmission routes. It is characterized as any instance in which non-intact skin, a mucous membrane, the eye, or direct contact with the bloodstream (such as through a tool puncture, needlestick, abrasion, or cut) comes into touch with the blood or any other substance that may possibly carry an infectious agent (such as saliva) [5,6]. Nosocomial infections present a clear and significant threat of bloodborne virus transmission to dental care workers, particularly dental interns who possess limited training in infection control protocols and often find themselves working on patients without help. The healthcare personnel most susceptible to NSIs are emergency department staff, surgeons, nurses, and laboratory technicians [7]. The utilization of needles is an inherent aspect of healthcare, and despite the presence of established protocols in hospitals regarding the appropriate management, as well as the use of safety-focused needle designs, needlestick accidents nevertheless occur more frequently in medical professionals, including surgeons and emergency room personnel. NSIs predominantly arise due to dangerous practices and excessive negligence exhibited by healthcare staff in the majority of instances [8].

The issue of NSIs gained significant attention in the healthcare field with the identification of HIV in the early 1980s. Since the implementation of universal precautions, there has been a significant reduction in the occurrence of NSIs. However, it is worth noting that these incidents still persist, albeit at a relatively low frequency. Currently, the primary concern following an NSI is not the transmission of HIV, but rather the potential acquisition of hepatitis B or hepatitis C [9,10].

The utilization of sharp instruments during dental procedures, along with the presence of saliva and blood, as well as the wide range of bacterial species found in the oral cavity, collectively contributes to the occupational risks associated with blood-borne infections in the dental setting. The prevention of NSIs poses a significant difficulty in nearly all healthcare settings. In the context of dental settings, the incidence of NSIs and SIs can be mitigated through adherence to established and well-recognized standard precautions aimed at preventing NSIs. Every healthcare facility must set up an infection control programme, which must be managed through an effective hospital infection prevention committee [11].

In our country, there is a lack of dependable surveillance data pertaining to occupational exposure. In order to build a robust infection control program, it is imperative to gather pertinent data regarding occupational exposure, illness prevalence, and associated factors [12-15]. This study aims to address the prevention and awareness of NSIs among dental students in Riyadh, Saudi Arabia. The objectives include evaluating the level of knowledge, attitudes, and perceptions regarding NSIs among dental students.

## Materials and methods

Research design

This cross-sectional study examined undergraduate dental students' knowledge, attitudes, and perceptions about NSIs throughout the 2023-2024 academic year in Riyadh, Kingdom of Saudi Arabia. Ethical approval (IRB/2577/23) and informed consent were collected from the study population. An online survey composed of previously tested and verified items served as the primary data collector. Participants were considered from various universities in their third and final years of undergraduate dental school in Riyadh. Students who were interested in participating were only included. However, to reduce the possibility of observer bias, undergraduate dentistry students were not allowed to take part in this study. Depending on the calculated sample size, a non-probability convenience sampling method was implemented. Twenty questions were included in the study's self-administered questionnaire, split evenly between demographic information and questions on respondents' familiarity with NSI. During normal sessions, the questionnaire was given out to eligible students in a way that protects their privacy by assigning them a random, unique identifier. After obtaining the responses from each of the participants, the responses were arranged in a database and were analyzed. Dental college and academic year were compared with respect to the occurrences and awareness regarding NSI among the participants. Other detailed findings have been represented and interpreted in the result section.

Inclusion and exclusion criteria

Inclusion criteria include dental undergraduates in their third and final years at various universities in Riyadh and students who were given informed consent for the study. This allows for a more comprehensive assessment of knowledge, attitudes, and perceptions, as students in these years are likely to have varying levels of exposure to clinical practice and infection control measures. Before participating, potential subjects were provided with a detailed information sheet that outlined the study's purpose, the nature of the survey, the expected duration, and potential risks or benefits. This document also emphasized their right to withdraw from the study at any point without consequences. Participants were then required to provide explicit written consent electronically through the online survey platform.

Exclusion criteria include undergraduate dental students performing research excluded from the study to minimize observer bias and dental students not in their third or final year not included in this study. Other academic years may have different educational experiences that could confound the results. The study pertains to dental care students in Riyadh, Saudi Arabia, thus participants from other universities were not included so as to maintain consistency in data collection and analysis.

Statistical analysis

Data was analyzed using Statistical Package for the Social Sciences (IBM SPSS Statistics for Windows, IBM Corp., Version 25.0, Armonk, NY) and we used descriptive statistics like frequencies and percentages for categorical data and mean, standard deviation, median, and interquartile range for continuous or ordinal data. The level of significance was determined using inferential statistics, including the Chi-square test, at 5%. Additional data analysis was done to acquire more information. This research design required improving healthcare safety and awareness among Riyadh dentistry students by identifying their NSI-related knowledge, attitudes, and behaviours. The level of significance was considered to be P<0.05.

## Results

The study comprised a total of 231 participants, with an average age of 23.53 years (±1.46), reflecting a relatively homogenous age distribution within the sample. Among the participants, there were 112 males and 119 females, indicating a fairly balanced gender representation (Table 1).

**Table 1:** Baseline information about the study participants

Parameter	Value
Age (years, Mean±Standard Deviation)	23.53±1.46
Gender	
Male	112
Female	119
Dental college you belong	
College 1	22
College 2	75
College 3	47
College 4	38
College 5	28
College 6	5
College 7	21
Academic Year	
Third Year	81
Fourth Year	149
Fourth Clinical Year	1

The participants were drawn from seven dental colleges in Riyadh, Saudi Arabia, which were anonymized as Colleges 1 through 7. These colleges were distributed across different regions of Riyadh, with Colleges 1 and 2 located in the East; Colleges 3 and 4 in the West; and Colleges 5, 6, and 7 in the Northern part of the city. Notably, the majority of participants were enrolled in College 2 (n=75) and College 3 (n=47), followed by College 4 (n=38), College 5 (n=28), College 1 (n=22), College 7 (n=21), and College 6 (n=5). This distribution provides a representation of students from various dental colleges in Riyadh, contributing to the diversity of the study sample.

Furthermore, the participants' academic years varied, with 149 in their fourth year of study, 81 in their third year, and one in their fourth Clinical Year. The predominance of fourth-year students likely reflects the larger enrollment in this academic year, which is a common pattern in dental education.

**Figure 1:**
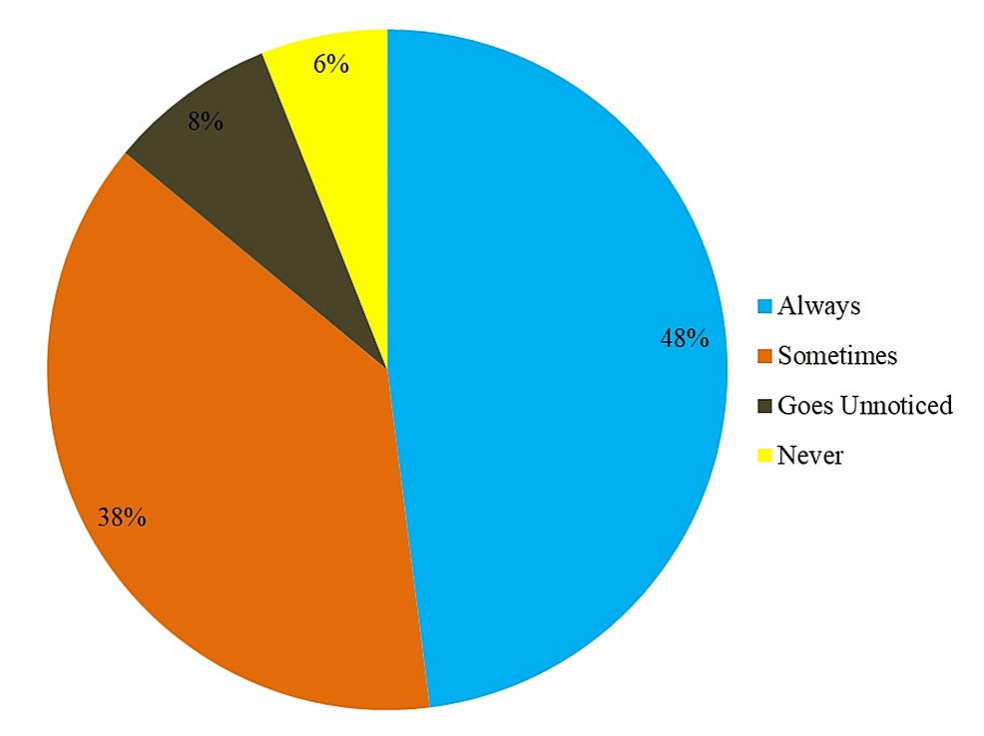
Frequency of needlestick injury as reported by the participants

Figure 1 shows the research participants' NSI frequency.

NSIs were reported by 48% of individuals as always, indicating a high incidence. A further 38% reported occasional NSIs, showing a large percentage of people. Only 8% said NSIs commonly go unrecognized, suggesting underreporting or lack of knowledge. Finally, 6% of subjects reported never having NSIs, which was a smaller portion. This chart shows NSI frequency in the study population.

**Figure 2:**
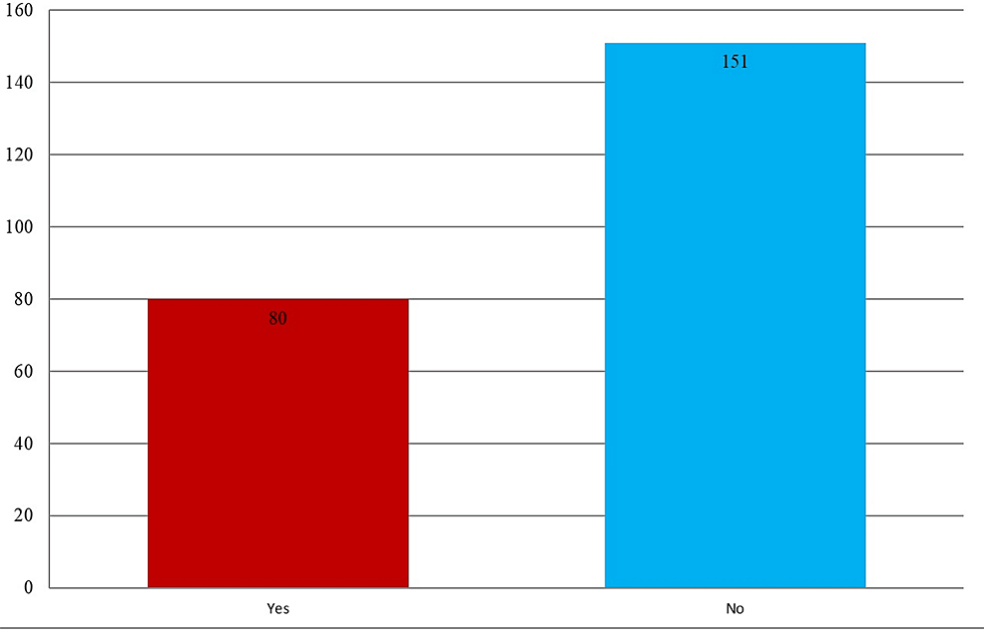
Occurrence of needlestick injury Units of measurement on the Y axis: Number of participants

Figure 2 shows the study participants' NSI reports, divided into two groups: Yes (n=80) and No (n=151).

The figure shows the dispersion clearly. NSIs were reported by 80 subjects and not by 151. This graph highlights the percentage of research participants who had NSIs and those who did not.

**Figure 3:**
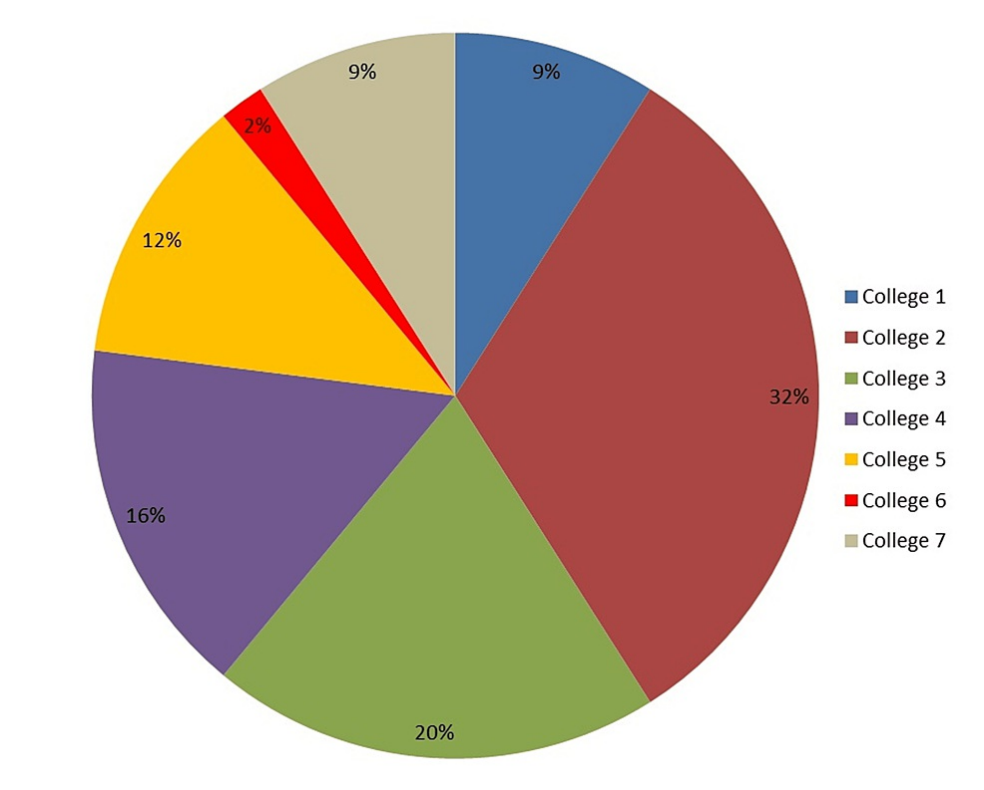
Percentage of participants who claimed to have a history of needlestick injury from each institution

Figure 3 shows the percentage of individuals with NSIs among institutions.

NSI reports were the greatest among the participants from College 2 (32%) and College 3 (20%). The lowest percentage (2%) of NSIs was observed in College 6 in the Northern part of Riyadh. Thus NSI experiences vary among individuals from different universities, revealing its prevalence across campuses.

Table 2 shows research participants' NSI knowledge and behaviours.

**Table 2:** Responses by the participants regarding the knowledge and attitude of needlestick injury

Queries	N	%
A number of participants know about universal/standard precaution guidelines	192	83.11
A number of participants who are aware of the diseases caused by needlestick injury	221	95.67
A number of participants use gloves while treating the patient	221	95.67
A number of participants who have habit of recapping the needle after injection	199	86.14
A number of participants who have been vaccinated against hepatitis B virus	220	95.23
A number of participants who have been provided with any instruction associated with the risk of bloodborne infection within the framework of your clinical training	188	81.38
A number of participants who think that the vaccination they have received will protect them from viral hepatitis	168	72.72
A number of participants think surgical gloves reduce the probability of needlestick injury by the wearer	109	47.18
A number of participants who agree that the needle should be recapped after use	215	93.07
A number of participants who are aware about postexposure prophylaxis	176	76.19

Most participants knew universal/standard precaution rules (83.11%), NSI illnesses (95.67%), and the significance of gloves during patient care. However, 86.14% recapped needles after injection, a dangerous practice. Most participants (95.23%) were vaccinated against hepatitis B and received bloodborne infection risk education during clinical training (81.38%). Notably, 72.72% claimed their vaccine prevented viral hepatitis. Only 47.18% believed surgical gloves lower NSI risk, and 93.07% believed needles should not be recapped. Additionally, 76.19% were aware of postexposure prophylaxis, indicating knowledge and practice gaps that should be addressed to improve healthcare safety.

Table 3 shows the study participants' institutions and academic years, as well as NSI contamination and illness awareness.

**Table 3:** The number of participants with respect to each institution NSI: needlestick injury

Colleges	Total number of students	Third year	Fourth year	Fourth year clinical	NSI contamination	Awareness of the diseases caused by NSI
College 1	22	11	11	0	9	18
College 2	75	21	53	1	17	72
College 3	47	7	40	0	21	44
College 4	38	18	20	0	10	38
College 5	23	15	8	0	10	23
College 6	5	1	4	0	3	5
College 7	21	8	13	0	10	21

Most participants were from College 2 with 75 students, mostly in their fourth year (53 students), 17 of whom reported NSI contamination and 72 knowledgeable of NSI disorders. College 3 had 47 participants, predominantly fourth-year students (40), 21 of them have NSI contamination and 44 know about NSI disorders. The various institutions included in this study experienced various student enrollments, NSI contamination, and awareness levels over academic years.

Table 4 shows research participants' clinical reactions to NSIs.

**Table 4:** Responses by the participants regarding perception towards needlestick injury

Queries	Number of participants	%
Frequency of needlestick injury reported		
Always	110	47.62
Sometimes	89	38.53
It goes unnoticed	18	7.79
Never	14	6.06
The commonest clinical activity to cause needlestick injury		
Recapping the needle	132	57.14
All of the above	82	35.50
Passing the instrument	9	3.90
Dental wiring Suturing	8	3.46
Type of measures taken after a needlestick injury		
Washing the site with soap water	78	33.77
Washing the site with water	76	32.90
Squeeze the blood	41	17.75
Washing the site with alcohol	36	15.58
The technique considered safe while recapping used needle		
Single-handed	210	90.91
Double-handed	21	9.09
Diseases that are transmitted by needlestick injury		
Hepatitis B; Hepatitis C; HIV	82	35.50
Hepatitis B	59	25.54
Hepatitis B; HIV	38	16.45
Hepatitis B; Hepatitis C; HIV; Tuberculosis	21	9.09
HIV	11	4.76
Hepatitis C	8	3.46
Hepatitis B; Hepatitis C	5	2.16
Hepatitis B; HIV; Tuberculosis	3	1.30
Hepatitis C; HIV	2	0.87
Hepatitis B; Hepatitis C; Tuberculosis	1	0.43
Hepatitis B; Tuberculosis	1	0.43

A total of 47.62% of subjects reported NSIs always, 38.53% occasionally, and 7.79% said they often go unreported. Recapping needles (57.14%) caused the most NSI; however, 35.50% said all clinical tasks were risky. Following an NSI, most people washed the site with soap and water (33.77%) or water alone (32.90%). Most (90.91%) said single-handed recapping was safe for used needles. Some participants knew that NSIs might spread hepatitis B, C, and HIV, while others did not. This result suggests that healthcare settings require better knowledge and safety to prevent and treat NSIs.

Table 5 presents the results of the statistical analysis of the association between NSI occurrence and awareness among students of various institutions.

**Table 5:** Association between NSI occurrence and awareness among students of various institutions *significant NSI: needlestick injury

College	NSI occurrence		χ² value	P-value	NSI awareness		χ² value	P-value
	Yes	No			Yes	No		
College 1	9	13	12.164	0.058	18	4	14.629	0.023*
College 2	17	58			72	3		
College 3	21	26			44	3		
College 4	10	28			38	0		
College 5	10	13			23	0		
College 6	3	2			5	0		
College 7	10	11			21	0		

While the Chi-square tests were used for comparisons indicating a statistically insignificant difference for NSI occurrence (χ²=12.164, p=0.058), NSI awareness (χ²=14.629, p=0.023) showed a statistically significant difference among students of various institutions.

Table 6 reveals that the academic year showed no significant relationship with either NSI occurrence (χ²=1.2, p=0.55) or NSI awareness (χ²=0.44, p=0.8).

**Table 6:** Association between NSI occurrence and awareness among students of various academic years NSI: needlestick injury

College	NSI occurrence		χ² value	P-value	NSI awareness		χ² value	P-value
	Yes	No			Yes	No		
Third year	31	50	1.20	0.55	60	21	0.44	0.80
Fourth year	49	100			108	41		
Fourth year clinical	0	1			1	0		

The statistical assessments conducted in this study, specifically the Kolmogorov-Smirnov (K-S) and Shapiro-Wilk tests, indicated that both the NSI occurrence and NSI awareness variables did not follow a normal distribution among the study participants. In other words, the data did not exhibit the typical bell-shaped curve that is often associated with normally distributed data. For NSI occurrence, both the Kolmogorov-Smirnov test (K-S Statistic=0.532, p<0.001) and the Shapiro-Wilk test (S-W Statistic=0.089, p<0.001) yielded statistically significant results. These results suggest that the distribution of NSI occurrences among the participants significantly deviated from a normal distribution. Similarly, for NSI awareness, both the Kolmogorov-Smirnov test (K-S Statistic=0.532, p<0.001) and the Shapiro-Wilk test (S-W Statistic=0.089, p<0.001) also produced statistically significant outcomes. This indicates that the distribution of NSI awareness levels among the participants significantly departed from a normal distribution. Furthermore, the study found a significant relationship between the occurrence of NSIs and awareness, indicating that increased awareness of various aspects related to NSIs could contribute to the prevention of such incidents among the participants or students. In practical terms, this means that efforts to enhance awareness and education regarding NSIs in the dental education setting may lead to a reduction in the occurrence of these injuries. The statistical tests showed that the data related to NSI occurrence and awareness were not normally distributed, emphasizing the need for interventions to improve awareness among dental students as a means to prevent NSIs effectively.

## Discussion

An NSI refers to an unintentional puncture wound caused by a hollow-bore needle that comes into contact with another individual's blood or bodily fluid, resulting in the penetration of the skin [14,15]. Healthcare workers (HCWs), especially dental professionals, have a potential occupational hazard of being exposed to blood-borne infections as a result of NSIs and other incidents involving SIs. It is imperative for HCWs to possess a comprehensive comprehension of the proper protocols for handling needles and sharps in order to establish a workplace atmosphere that is devoid of potential hazards [15].

The objective of the conducted study was to evaluate the knowledge, attitude, and perception of NSIs among dental students in various dental colleges located in Riyadh. Dental students lack adequate awareness about NSIs as well as how to prevent them, thus it is necessary to train them in the use of safety equipment, post-exposure prophylaxis procedures, and universal precaution guidelines. This will help to prevent NSIs from occurring in the future [14-16].

Throughout the year, a significant number of HCWs continue to be vulnerable to various life-threatening diseases, including those transmitted through blood. NSIs are widely recognized as a prevalent occupational health risk within the healthcare industry. By integrating these strategies, dental education can become safer for students, faculty, and patients alike, ultimately contributing to improved HCW safety and the overall quality of dental care in Riyadh, Saudi Arabia. Dental practitioners face an increased susceptibility to NSIs as a result of the confined and constrained nature of their work environment. A comprehensive literature study was conducted to ascertain the level of knowledge, awareness, and adherence to NSI protocols among dental professionals and students in Riyadh. The level of knowledge and awareness exhibited by dental students is deemed satisfactory, albeit with notable disparities in the implementation and handling of NSI across several research studies. There exists a necessity for further research endeavors encompassing dental specialists [15-17].

A previous study was undertaken to assess potential risk factors for NSIs suffered by undergraduate nursing as well as dentistry students enrolling at King's College London's (KCL) Dentistry Institute [16]. A review of the past was conducted to assess the incident reports pertaining to NSIs that were recorded throughout a span of two years. Several factors can influence the occurrence of injuries during the administration of local anesthetic (LA) in dental departments. These factors include the specific department, the study year of the dental professional, and the timing of the injury in relation to activities such as recapping a conventional syringe, cleaning a work area, or during the disposal process [17]. The utilization of safety syringes has been found to result in a decrease in the number of NSIs. All NSIs will be completely eliminated by enforcing a non-recapping policy and promptly disposing of either conventional or safe syringe devices after injection caused by clearance activities among nurses. In order to mitigate the occurrence of NSIs, education assumes a crucial role, particularly in the successful execution of the transition to safety syringes, accompanied by comprehensive training programs [15-18].

NSIs pose a significant risk to dental interns. The study aimed to investigate the occurrence and attributes of NSIs among dental interns in their initial year of clinical training [18]. Additionally, the study sought to analyze potential factors that contribute to the risk of NSIs and to analyze the patterns of reporting such incidents. Dental trainees are vulnerable to NSIs during their first year of clinical experience. Significant emphasis should be placed on the careful consideration of dental burs, syringe needles, ultrasonic chips, and suture needles. The absence of chairside support poses a significant risk in relation to NSIs. There is a need for improvement in the training provided to first-year dental interns in the area of chairside assistance. It is imperative for dental interns in their first year of training to enhance their understanding of overlooked behaviors associated with NSIs [19].

NSIs are more common among dental students. The epidemiology of needlesticks as well as SIs amongst dental school pupils in China, however, is poorly documented in the literature. Another study determined the incidence of needlesticks and SIs at a renowned dental training facility in China and identified any factors that might be associated with these types of incidents. It has been established that dentistry students are vulnerable to injuries from needlesticks. The data unequivocally shows that dental students need to have a deeper understanding of NSI prevention. It is impossible to exaggerate the significance and requirement of infection prevention and control teaching at dental institutions since it is essential for reducing the likelihood of needlesticks as well as SIs [18-20].

NSIs represent the most prevalent means via which bloodborne viruses and/or diseases, such as HIV, hepatitis C, and hepatitis B, can spread from patient to HCWs. Dental students are susceptible to illnesses and injuries as a result of inadvertent contamination during their occupational exposure in practical settings. Limited data exists about the awareness and experiences of National Service Indemnity among dentistry students in Kenya [18]. A research investigation was undertaken to ascertain the level of knowledge and experiences pertaining to NSI among dentistry students enrolled at the University of Nairobi Dentistry Hospital (UONDH). Despite a high level of awareness of the possibility of cross-infection resulting from NSI, there was a notable decline in understanding regarding the methods of prevention and adherence to established protocols [19-22].

The study further inferred that most often after an NSI occurs, students wash the site with soap water and recap the needle after use, causing more NSI than other activities. This shows that proper training is required to be given to the students before handling needle-related activities. The survey also found that there are some discrepancies of thoughts among the students like half of them do not believe that the gloves can prevent NSI, which needs to be properly addressed by the guide or the professor during the class. The survey also concluded that there is a need to design proper training as 38% of the students have experience with NSI which is quite a high number. Only 35.5% of the students responded that hepatitis B, C, and HIV get transmitted through NSI while 25.54% responded that only hepatitis B gets transmitted. This shows that there is inadequate knowledge among the students regarding the transmission of disease. This study brought forward the facts and figures based on which, awareness or teaching programmes can be arranged so that the students experimenting or using needlesticks, should be kept well informed about the hazards of NSI.

The implications of this study extend to dental education globally, emphasizing the importance of rigorous infection control training. Dental colleges and policymakers should focus on tailored education, regular assessments, a culture of reporting, collaboration with healthcare authorities, and the integration of infection control experts to foster a safer environment for dental students and uphold the highest standards of infection control in dental education.

The study limitations include firstly, our sample was drawn from selected universities in Riyadh, potentially limiting the generalizability of the results to the broader population of dental students in Saudi Arabia. Additionally, relying on self-reported data through an online survey introduces the potential for response bias, where participants may provide socially desirable or inaccurate responses. However, this survey study may have some questionnaire bias and should be conducted with more students. The authors suggest that this kind of survey should be conducted from time to time to get an update on the awareness of the students regarding NSI. Finally, external factors that may have influenced knowledge and attitudes before or after the study period were not considered. Despite these limitations, our study contributes valuable insights into the awareness of NSIs among dental students and underscores the need for ongoing efforts to enhance education and awareness in dental education settings. Tailoring educational interventions to specific dental colleges can be an effective strategy to improve infection control practices. While our study focused on dental students in Riyadh, the implications extend to dental education globally, emphasizing the importance of rigorous infection control training. By addressing these issues, dental education institutions can play a pivotal role in ensuring the safety of dental professionals and the quality of patient care.

## Conclusions

The study has concluded that NSI occurrence significantly depends on the awareness among the participants. This implies that the occurrences reduce with increased awareness among the participants. The study also showed various important findings. The study concluded that practical skills and awareness need to be increased more among the students to prevent NSI in the universities and colleges. There is no such correlation between reduced NSI occurrence or awareness with that of the academic year. So, it implies that awareness has a positive impact on NSI occurrence (that is, reduced NSI occurrence) while both are irrespective of the academic year of the students.

Therefore, dental colleges and policymakers should focus on tailored education, regular assessments, a culture of reporting, collaboration with healthcare authorities, and the integration of infection control experts to foster a safer environment for dental students and uphold the highest standards of infection control in dental education.
